# Immunization with an *Escherichia coli*-Produced Hepatitis E Virus Genotype 3 Virus-like Particle (HEV-3_p495) Confers Protection Against HEV-3 and HEV-4 Challenge in Pigs

**DOI:** 10.3390/v18101122

**Published:** 2026-10-10

**Authors:** Satoshi Kunita, Masaharu Takahashi, Tominari Kobayashi, Kazumoto Murata, Hiroaki Okamoto

**Affiliations:** 1 Center for Experimental Medicine, Jichi Medical University School of Medicine, 3311-1 Yakushiji, Shimotsuke 329-0498, Tochigi, Japan; 2 Division of Virology, Department of Infection and Immunity, Jichi Medical University School of Medicine, 3311-1 Yakushiji, Shimotsuke 329-0498, Tochigi, Japan; mtaka84@jichi.ac.jp (M.T.); kobayashi.tominari@jichi.ac.jp (T.K.); kmurata@jichi.ac.jp (K.M.)

**Keywords:** hepatitis E virus, virus-like particle, HEV-3_p495, pig, HEV-3, HEV-4, cohabitation

## Abstract

Hepatitis E virus (HEV) genotypes 3 and 4 are major zoonotic pathogens, with domestic pigs serving as an important reservoir. Vaccination of pigs may therefore help reduce HEV circulation and zoonotic transmission. We evaluated the protective efficacy of an *Escherichia coli*-produced HEV-3 virus-like particle (VLP), HEV-3_p495, against homologous HEV-3 and heterologous HEV-4 challenge in pigs. Juvenile pigs vaccinated three times with either 80 or 400 μg of HEV-3_p495 remained negative for HEV RNA in serum and feces during prolonged cohabitation with naturally HEV-3-infected pigs, whereas all non-vaccinated controls became infected. At market age, vaccination also provided strong protection against intravenous HEV-3 challenge, with complete protection against detectable infection in the four-dose group and in two of three pigs in the three-dose group. Importantly, HEV-3_p495 conferred cross-protection against HEV-4 following cohabitation exposure. Two-dose vaccination prevented detectable infection in two of three young pigs and delayed fecal virus detection without viremia in the remaining pig. At market age, all vaccinated pigs remained negative for HEV RNA in serum and feces following HEV-4 exposure, whereas all non-vaccinated controls became infected. These findings provide proof-of-concept evidence for homologous and cross-genotype protection by HEV-3_p495 and support its further evaluation as a candidate swine vaccine for reducing HEV infection and shedding.

## 1. Introduction

Hepatitis E virus (HEV) is a major cause of acute viral hepatitis worldwide and an important zoonotic pathogen. According to the World Health Organization (WHO), an estimated 19.47 million acute HEV infections occur annually, resulting in more than 3450 deaths in 2021 [1]. HEV is also recognized as one of the viruses with the highest risk of animal-to-human spillover and as a leading foodborne pathogen in recent joint assessments by WHO and the Food and Agriculture Organization (FAO) [2,3], underscoring its importance to both public health and food safety.

Although most HEV infections are asymptomatic or self-limiting, severe disease may occur in vulnerable populations. Pregnant women and patients with pre-existing liver disease are at increased risk of acute liver failure, whereas immunocompromised individuals, particularly solid organ transplant recipients, may develop chronic hepatitis that can progress to cirrhosis [4,5,6,7,8]. In addition, a broad spectrum of extrahepatic manifestations, including neurological, renal, and hematological disorders, has been increasingly recognized [9,10].

HEV belongs to *Paslahepevirus balayani* (formerly *Orthohepevirus A*) and comprises eight human-infecting or potentially human-infecting genotypes (HEV-1 to HEV-8) [11,12]. The virus circulates as quasi-enveloped particles in the bloodstream but is shed as non-enveloped particles in feces [13,14,15]. Its approximately 7.2-kb positive-sense, single-stranded RNA genome contains three major open reading frames (ORF1, ORF2, and ORF3), encoding proteins required for viral replication, capsid formation, and virion egress and virus–host interactions, respectively [16,17,18]. Molecular genotyping and subtyping are indispensable for epidemiological surveillance and source attribution [19].

Among the eight genotypes, HEV-1 and HEV-2 infect only humans and are transmitted primarily through contaminated drinking water, causing large outbreaks in resource-limited settings. In contrast, HEV-3 and HEV-4 infect both humans and several mammalian species, particularly domestic pigs and wild boars, and are responsible for zoonotic foodborne infections associated with contaminated pork products. HEV-3 is widely distributed worldwide, including Japan, North America, and Europe, whereas HEV-4 predominates in East and Southeast Asia. Domestic pigs constitute the principal reservoir of zoonotic HEV, and numerous molecular epidemiological studies have demonstrated close genetic relationships between human and swine isolates, highlighting the importance of controlling HEV infection in pig populations to reduce foodborne transmission to humans [19,20,21,22].

Because the ORF2 capsid protein contains highly conserved neutralizing epitopes, recombinant ORF2 proteins have long been regarded as promising vaccine candidates. A baculovirus-expressed recombinant HEV-1 ORF2 vaccine developed by GlaxoSmithKline demonstrated approximately 95% efficacy in a phase II clinical trial but was not commercialized [23]. Subsequently, the HEV 239 vaccine, consisting of a 239-amino-acid fragment of the ORF2 capsid protein derived from a genotype 1 HEV strain and produced in *Escherichia coli*, demonstrated > 90% efficacy against clinically apparent hepatitis E in a large phase 3 trial [24]. Extended follow-up initially demonstrated sustained protection for at least 4.5 years [25], and a subsequent 10-year follow-up study further confirmed durable vaccine efficacy against clinically apparent hepatitis E [26]. Notably, although HEV 239 is derived from genotype 1, most genotype-confirmed hepatitis E cases in the phase 3 trial were caused by genotype 4 HEV, supporting cross-genotype protection against clinically apparent disease. However, these clinical trials primarily evaluated clinically apparent hepatitis E rather than asymptomatic HEV infection and therefore do not establish complete protection against infection. Whether similarly broad protection can be achieved against infection itself, particularly in the natural swine reservoir, remains an important question.

In contrast to human vaccination, relatively little attention has been paid to vaccination of pigs, the principal reservoir of zoonotic HEV-3 and HEV-4. Preventing infection in the natural host could reduce viral circulation in swine populations and consequently decrease the risk of foodborne transmission to humans [22,27,28]. However, only a limited number of vaccination–challenge studies have been conducted in pigs. Previous vaccination studies in pigs using recombinant HEV capsid antigens or VLP-based vaccines have demonstrated varying degrees of protection, ranging from reductions in viremia and fecal virus shedding to prevention of detectable infection under some experimental conditions [29,30,31,32]. However, complete protection has not been consistently achieved, and direct evidence that an HEV-3-derived vaccine can protect pigs against heterologous HEV-4 infection remains limited.

Here, we evaluated the protective efficacy of an *E. coli*-derived HEV-3 VLP vaccine (HEV-3_p495) [33] against experimental challenge with both HEV-3 and HEV-4 in pigs. This study was designed to determine whether vaccination could achieve complete protection against infection in the natural host and thereby provide a practical strategy for reducing zoonotic HEV transmission.

## 2. Materials and Methods

### 2.1. Experimental Animals

**Figure 1:**
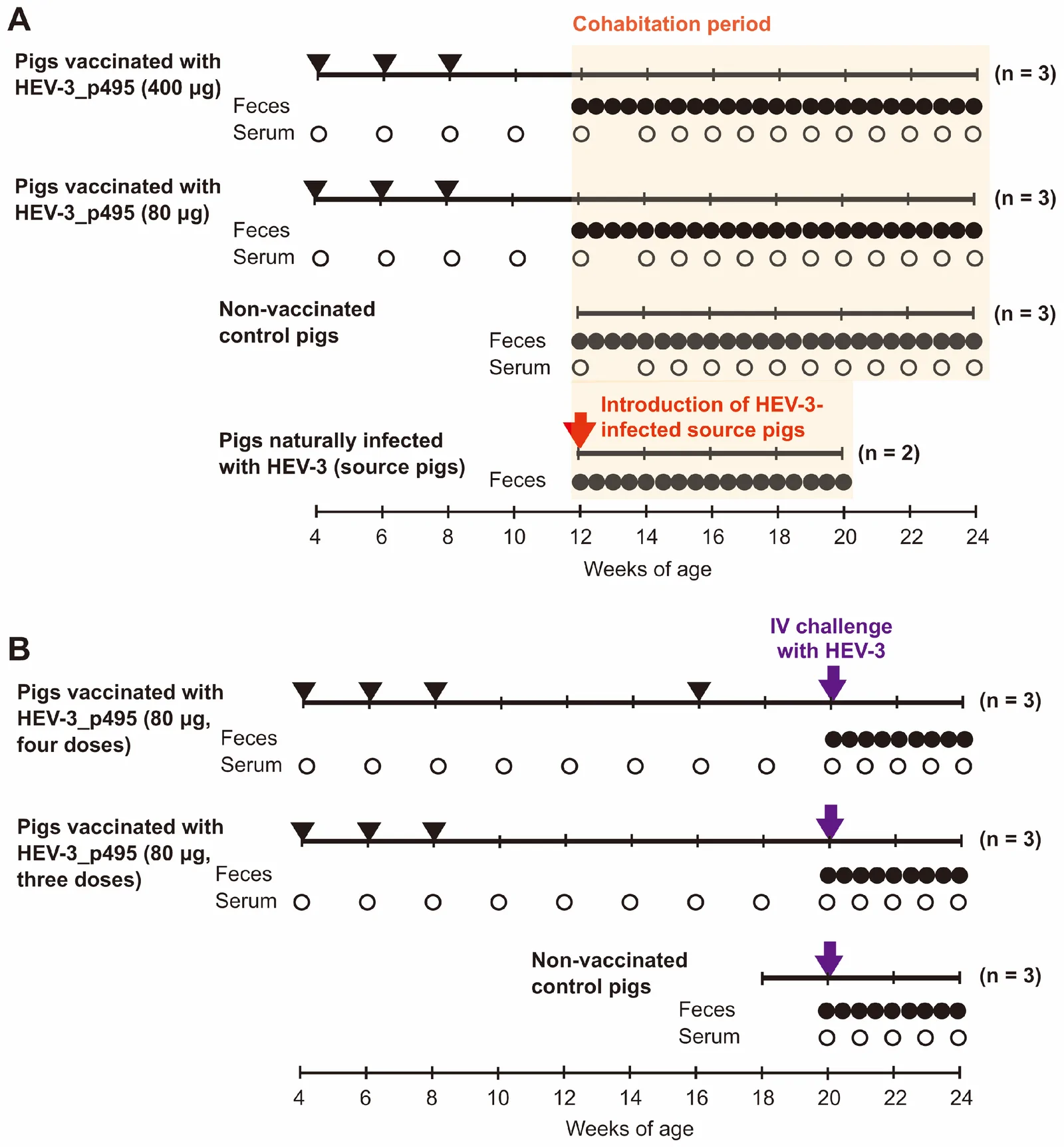

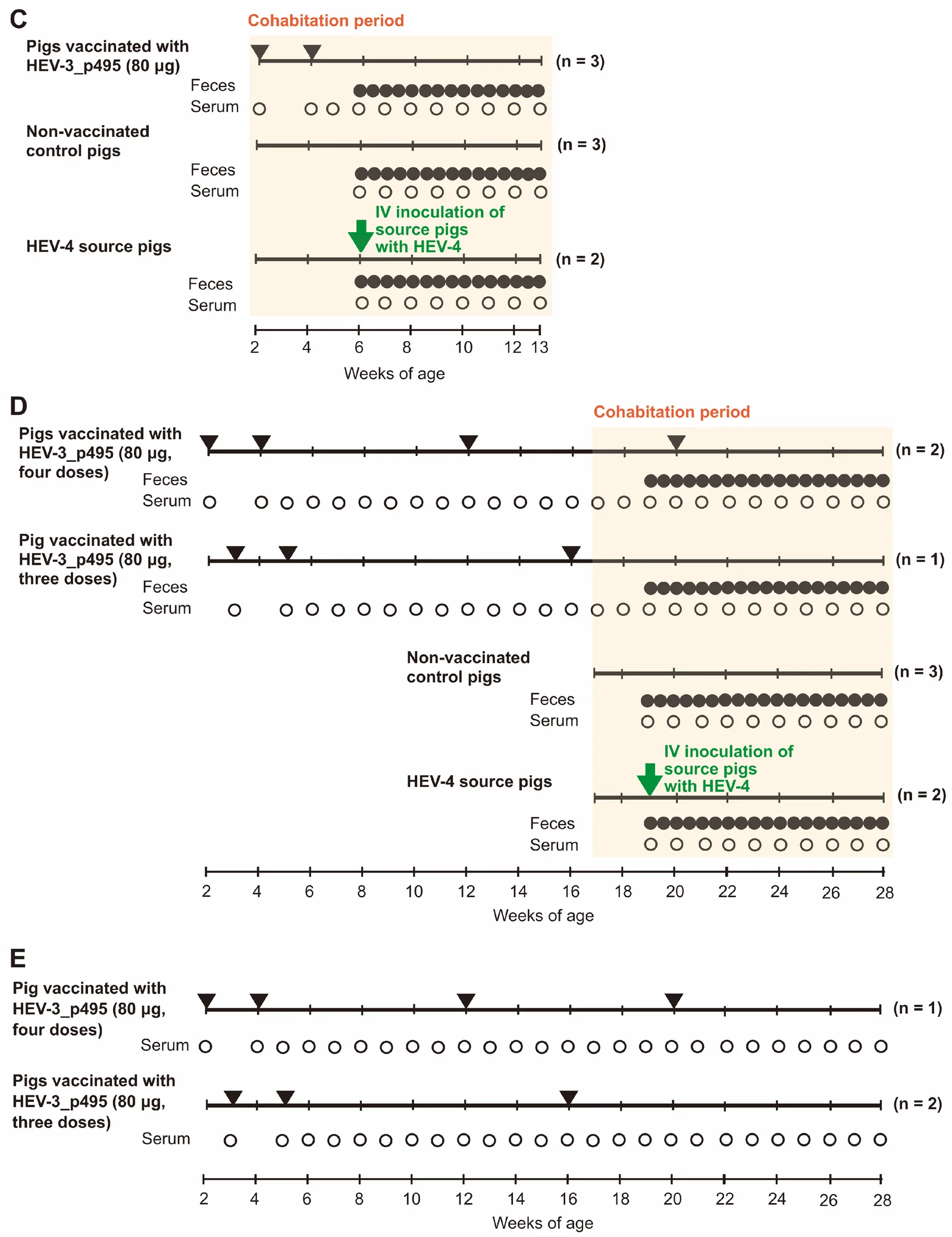
Experimental design of vaccination and HEV challenge experiments in pigs. (**A**) Experimental design for the juvenile-stage homologous HEV-3 cohabitation challenge. Pigs were intramuscularly immunized with either 400 or 80 μg of the HEV-3 VLP vaccine (HEV-3_p495) at 4, 6, and 8 weeks of age (n = 3 per group). At 12 weeks of age, the six vaccinated pigs were co-housed with three non-vaccinated pigs serving as controls and two pigs naturally infected with HEV-3 in a group-housing pen. All 11 pigs were co-housed until 20 weeks of age to establish a natural fecal–oral transmission model. The two source pigs were then removed, and the remaining nine pigs were co-housed and monitored until 24 weeks of age. (**B**) Experimental design for the market-age homologous HEV-3 challenge. Pigs received 80 μg of the HEV-3_p495 vaccine at 4, 6, 8, and 16 weeks of age (four-dose group, n = 3) or at 4, 6, and 8 weeks of age (three-dose group, n = 3). Non-vaccinated pigs served as controls (n = 3). All nine pigs were raised separately and intravenously challenged at 20 weeks of age with 10^4^ genome copies of the HEV-3 swEJM2100729F strain. (**C**) Experimental design for the early-age heterologous HEV-4 cohabitation challenge. Cohabitation of eight pigs was initiated at 2 weeks of age in a group-housing pen. Three pigs received 80 μg of the HEV-3_p495 vaccine at 2 and 4 weeks of age, while three non-vaccinated pigs served as controls. At 6 weeks of age, while cohabitation was continued, the source pigs were intravenously inoculated with HEV-4 to initiate the cohabitation challenge. (**D**) Experimental design for the market-age heterologous HEV-4 cohabitation challenge. Two pigs received four doses of the HEV-3_p495 vaccine (80 μg) at 2, 4, 12, and 20 weeks of age, and one pig received three doses at 3, 5, and 16 weeks of age. At 17 weeks of age, the three vaccinated pigs were co-housed with three non-vaccinated control pigs and two HEV-4 source pigs. At 19 weeks of age, while cohabitation was continued, the source pigs were intravenously inoculated with HEV-4 to initiate the cohabitation challenge. (**E**) Vaccination and serum-sampling schedules for additional pigs used to achieve a total of three animals per vaccination regimen in the market-age HEV-4 challenge study. One pig received the four-dose regimen (80 μg at 2, 4, 12, and 20 weeks of age), and two pigs received the three-dose regimen (80 μg at 3, 5, and 16 weeks of age). All three pigs were raised separately. Black arrows indicate HEV-3_p495 vaccination, and pink-shaded areas indicate the periods of cohabitation. Black and open circles indicate fecal and serum sampling, respectively. Colored arrows indicate the introduction of HEV-3-infected source pigs (**A**) or intravenous inoculation of HEV-3 (**B**) or HEV-4 (**C**,**D**), as indicated. IV, intravenous; HEV, hepatitis E virus.

All animal experiments were approved by the Institutional Animal Care and Use Committee of Jichi Medical University (Approval Nos. 21025-01, 22014-01, 23015-02, and 25018-01). A total of 37 specific pathogen-free pigs aged 2, 4, 12, 17, or 18 weeks were obtained from an HEV-negative farm (Sanesu Breeding Co., Ltd., Chiba, Japan). Before enrollment, all pigs were confirmed to be free of HEV infection by testing for serum anti-HEV IgG antibodies and HEV RNA, as described below. In addition, two 12-week-old pigs naturally infected with the HEV-3 swEJM2100729F strain [34] were used as source animals in the HEV-3 cohabitation challenge experiments (Figure 1A). All pigs were housed in biosafety-controlled animal facilities (Center for Development of Advanced Medical Technology, CDAMTec, Jichi Medical University, Shimotsuke, Japan; NAS Laboratory Co., Ltd., Narita, Japan) under standard husbandry conditions with *ad libitum* access to feed and water.

### 2.2. Experimental Design

Two independent experiments were conducted to evaluate the protective efficacy of the HEV-3_p495 vaccine against homologous HEV-3 and heterologous HEV-4 challenge in pigs. Experiment I assessed protection against HEV-3 challenge at the juvenile and market stages, whereas Experiment II evaluated cross-protective efficacy against HEV-4 challenge using different vaccination schedules.

**Experiment I (Protective efficacy against homologous HEV-3 challenge):** Experiment I consisted of two independent challenge studies designed to evaluate vaccine efficacy at different stages of pig production. The first study employed a cohabitation challenge initiated at 12 weeks of age to assess protection during the juvenile stage (Figure 1A), whereas the second used an intravenous challenge at 20 weeks of age to evaluate protection at market age (Figure 1B).

For the juvenile-stage study, piglets were randomly assigned to three groups (n = 3 per group). Pigs in the 400-μg group received intramuscular immunizations with 400 μg of the HEV-3_p495 vaccine at 4, 6, and 8 weeks of age, whereas those in the 80-μg group received 80 μg according to the same schedule. These six vaccinated pigs were raised individually until 12 weeks of age. At 12 weeks of age, they were placed in a group-housing pen with three non-vaccinated control pigs and two HEV-3 source pigs (introduced from an HEV-3-positive farm and confirmed to be shedding HEV in their feces). All 11 pigs were co-housed until 20 weeks of age to establish a natural fecal–oral transmission model, at which point the two source pigs were removed from the pen. The six vaccinated pigs and the three control pigs were co-housed until 24 weeks of age (Figure 1A). Fecal and serum samples were obtained as indicated in Figure 1A.

For the market-age study, piglets were allocated to three groups (n = 3 per group). Pigs in the four-dose group were administered 80 μg of the HEV-3_p495 vaccine at 4, 6, 8, and 16 weeks of age, whereas those in the three-dose group received 80 μg of the vaccine at 4, 6, and 8 weeks of age. Pigs in the non-vaccinated group served as controls. All nine pigs were raised separately throughout the study. At 20 weeks of age, all pigs were intravenously challenged with 10^4^ genome copies of the HEV-3 swEJM2100729F strain in phosphate-buffered saline containing 0.2% bovine serum albumin (0.2% BSA-PBS), corresponding to a 10,000-fold dilution of a 15% fecal suspension (Figure 1B). The challenge dose was selected based on preliminary experiments in which all pigs became infected at each of the tested inoculum doses (10^3^, 10^4^, and 10^5^ genome copies; Supplementary Table S1). Fecal and serum samples were collected according to the schedule shown in Figure 1B.

**Experiment II (Protective efficacy against heterologous HEV-4 challenge):** Experiment II was designed to optimize the vaccination regimen and to determine whether the HEV-3_p495 vaccine confers cross-protection against heterologous HEV-4 HE-JF5/15F strain challenge. Initially, a two-dose vaccination regimen completed immediately after weaning, with immunizations administered at 2 and 4 weeks of age, was evaluated using a cohabitation model in which source pigs were inoculated at 6 weeks of age. Three pigs of the same age served as a non-vaccinated control (Figure 1C). Protective efficacy was assessed by monitoring virological and serological responses following challenge as indicated in Figure 1C.

To evaluate the durability of vaccine-induced immunity and the effect of booster immunization, additional vaccination schedules were examined in a market-age challenge model. Two pigs received booster immunizations at 12 and 20 weeks of age following the primary immunizations at 2 and 4 weeks of age, resulting in a four-dose schedule (2, 4, 12, and 20 weeks), whereas one pig received a three-dose schedule consisting of vaccinations at 3, 5, and 16 weeks of age (Figure 1D). These pigs were co-housed with the non-vaccinated control pigs and the HEV-4 source pigs from 17 weeks of age; the source pigs were intravenously inoculated with HEV-4 at 19 weeks of age (Figure 1D).

To obtain three animals per vaccination schedule for descriptive comparison of anti-HEV IgG responses, three additional pigs received the corresponding immunization schedules but were not subjected to HEV challenge (Figure 1E).

### 2.3. Inoculum and Challenge Procedures

The inocula used for intravenous challenge consisted of 15% (*w*/*v*) fecal suspensions containing the HEV-3 swEJM2100729F strain, which has previously been demonstrated to be infectious in cultured cells [34]. A preliminary study confirmed that intravenous inoculation with 10^3^–10^5^ genome copies established HEV-3 infection in all inoculated pigs; therefore, 10^4^ genome copies were selected for the market-age challenge (Supplementary Table S1).

To establish a challenge model that closely mimics natural HEV transmission under field conditions, a cohabitation challenge model was employed using HEV-infected source pigs. Pigs naturally infected with the HEV-3 swEJM2100729F strain, obtained from an HEV-positive farm [34], or those experimentally infected with the HEV-4 HE-JF5/15F strain [35] by intravenous inoculation with 10^6^ genome copies of the corresponding strain in 0.2% BSA-PBS, corresponding to a 26-fold dilution of a 15% fecal suspension, served as source animals. Experimental pigs were co-housed with the infected source pigs to allow fecal–oral transmission under controlled conditions. This model enabled reproducible evaluation of the protective efficacy of the HEV-3_p495 vaccine against naturally transmitted HEV infection.

### 2.4. Vaccine and Immunization

The N- and C-terminally truncated HEV-3 VLP vaccine, HEV-3_p495 (amino acids 112–606), expressed in *E. coli* [33], was used throughout this study. Immediately before administration, the vaccine was mixed with Alhydrogel^®^ adjuvant (Croda Japan K.K., Tokyo, Japan) at a volume ratio of 4:1. All immunizations were administered by intramuscular injection into the upper neck region just behind the ear.

### 2.5. Sample Collection and Processing

Serum and individual rectal fecal samples were collected at regular intervals throughout each challenge experiment, as illustrated in Figure 1A–E. Liver samples were collected at necropsy from vaccinated pigs in the market-age HEV-3 challenge, early-age HEV-4 challenge, and market-age HEV-4 challenge experiments. Liver samples were not available from the vaccinated pigs in the juvenile-stage HEV-3 cohabitation experiment. Fecal suspensions (15%, *w*/*v*) were prepared as previously described [36]. Briefly, 5 g of each fecal sample was suspended in phosphate-buffered saline (PBS; pH 7.2; Thermo Fisher Scientific Inc., Waltham, MA, USA) to a final concentration of 15% (*w*/*v*). The suspensions were centrifuged at 1600× *g* for 30 min at 4 °C using a refrigerated centrifuge (Eppendorf Himac Technologies Co., Ltd., Ibaraki, Japan), and the resulting supernatants were collected. The supernatants were subsequently clarified by centrifugation at 6200× *g* for 5 min at 4 °C using a refrigerated microcentrifuge (Tomy Seiko Co., Ltd., Tokyo, Japan). Liver samples were aliquoted immediately after collection, and the liver aliquots, serum samples, and clarified fecal supernatants were stored at −80 °C until further analysis.

### 2.6. Enzyme-Linked Immunosorbent Assay for Detection of Anti-HEV IgG Antibodies

Serum anti-HEV IgG antibodies were measured by enzyme-linked immunosorbent assay (ELISA) as previously described [37]. Serum samples were diluted 1:100 and tested using purified recombinant ORF2 protein derived from the HEV-4 HE-J1 strain, expressed in silkworm pupae [38], as the coating antigen.

An optical density (OD) value of 0.274 measured at 450 nm was used as the cutoff for seropositivity. Samples with OD values greater than or equal to the cutoff were considered positive for anti-HEV IgG antibodies. The specificity of positive reactions was confirmed by an absorption assay using the same recombinant ORF2 protein as the coating antigen at a final concentration of 50 μg/mL, as previously described [37]. Samples showing a reduction of ≥70% in OD value following absorption with the recombinant ORF2 protein were regarded as specifically positive for anti-HEV IgG antibodies.

### 2.7. Qualitative and Quantitative Detection of HEV RNA

All serum samples and 15% (*w*/*v*) fecal suspensions collected throughout the study, as well as available liver samples collected at necropsy, were tested for the presence of HEV RNA. Total RNA was extracted from 100 μL of each serum or fecal sample using TRIzol LS Reagent (Thermo Fisher Scientific Inc.) according to the manufacturer’s instructions. For liver samples, 50 mg of each liver specimen was homogenized in TRIzol Reagent (Thermo Fisher Scientific Inc.), and total RNA was extracted as previously described [21]. Extracted RNA was reverse-transcribed using SuperScript IV (Thermo Fisher Scientific Inc.). Qualitative detection of HEV RNA was performed by nested polymerase chain reaction (PCR) targeting the overlapping region of ORF2 and ORF3 (ORF2/3-143 PCR), as previously described [21]. PCR amplification was carried out using TaKaRa Ex Taq (TaKaRa Bio Inc., Shiga, Japan). The expected amplicon sizes were 176 base pairs (bp) for the first-round PCR and 143 bp for the second-round PCR.

HEV RNA was quantified by real-time reverse transcription-PCR (real-time RT-PCR) as previously described [39]. Briefly, total RNA extracted from 100 μL of each serum or fecal sample was analyzed using the QuantiTect Probe RT-PCR Kit (Qiagen, Tokyo, Japan) on a LightCycler instrument (Roche Diagnostics K.K., Tokyo, Japan). Reverse transcription was performed at 50 °C for 20 min, followed by an initial denaturation step at 95 °C for 15 min and 45 cycles of amplification consisting of denaturation at 95 °C for 1 s and annealing/extension at 60 °C for 60 s. Each sample was analyzed in duplicate, and the mean value was used for subsequent analyses. The lower limit of detection of the real-time RT-PCR assay was 480 genome copies/g of feces.

### 2.8. Evaluation of Protective Efficacy

The protective efficacy of the HEV-3_p495 vaccine was evaluated by comparing vaccinated and non-vaccinated pigs with respect to the incidence and duration of viremia, the incidence and duration of fecal virus shedding, peak viral loads in serum and fecal samples, and the kinetics and magnitude of anti-HEV IgG antibody responses. These parameters were used to assess the ability of the vaccine to prevent or attenuate homologous HEV-3 challenge and heterologous HEV-4 challenge at both the juvenile and market stages.

### 2.9. Statistical Analysis

Anti-HEV IgG levels in serum are expressed as OD values and are presented as the mean ± standard deviation (SD). Because of the small number of animals and differences in vaccination timing between the two schedules, the data are presented descriptively without inferential statistical comparisons. Individual values are provided in Supplementary Table S2.

## 3. Results

### 3.1. Protective Efficacy of Three-Dose HEV-3_p495 Vaccination Against Homologous HEV-3 Infection Following Cohabitation Challenge in Juvenile Pigs

**Figure 2:**
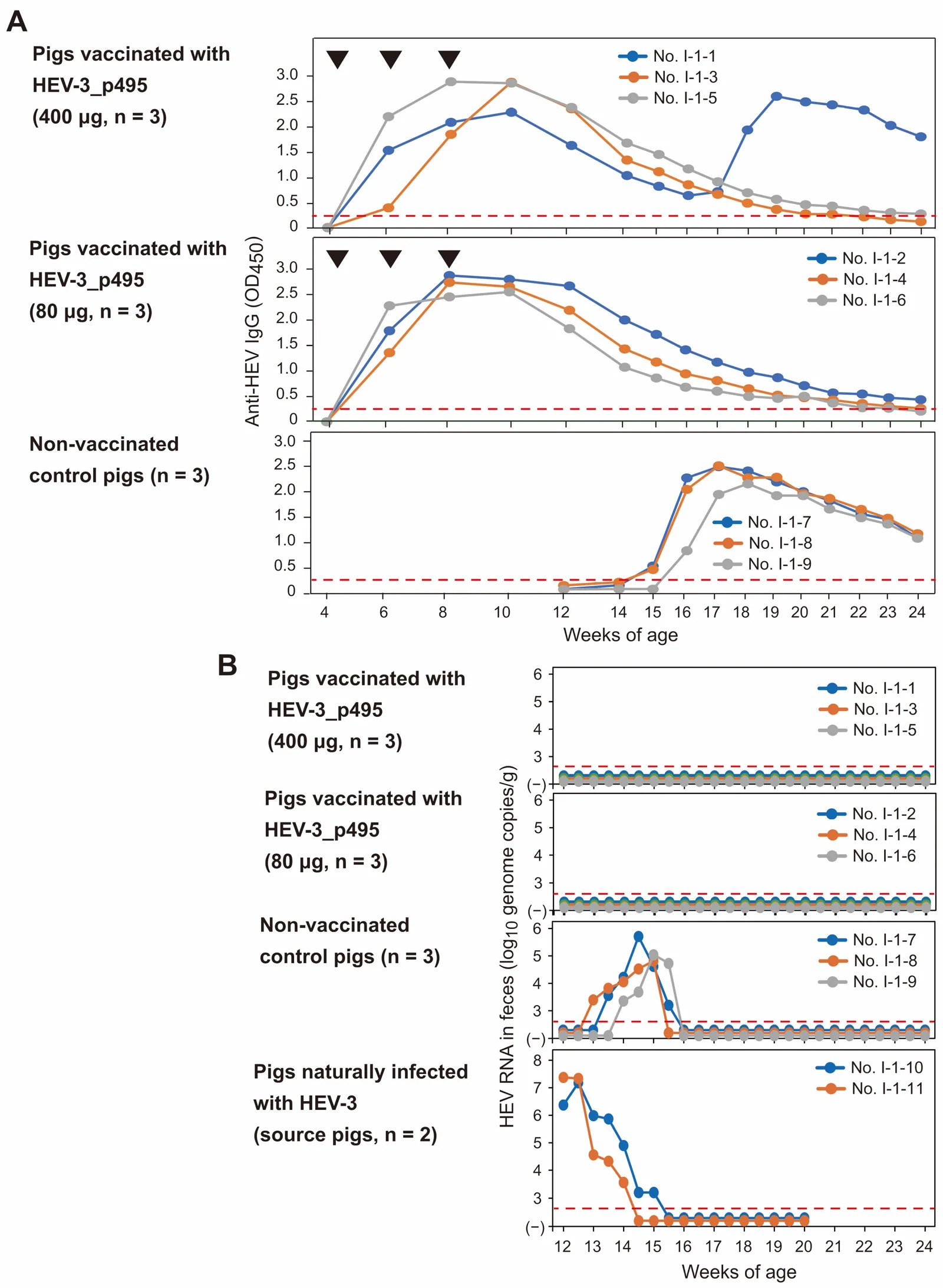
Anti-HEV IgG responses and fecal HEV RNA levels following homologous HEV-3 cohabitation challenge in juvenile pigs. (**A**) Anti-HEV IgG responses in pigs vaccinated with 400 or 80 μg of the HEV-3_p495 vaccine (n = 3 per group) and non-vaccinated control pigs (n = 3). Pigs were vaccinated at 4, 6, and 8 weeks of age and subsequently co-housed with two pigs naturally infected with HEV-3 (source pigs) from 12 weeks of age, as shown in Figure 1A. Anti-HEV IgG levels in serum are expressed as OD450 values. (**B**) HEV RNA levels in feces from the vaccinated pigs, non-vaccinated control pigs, and HEV-3 source pigs. HEV RNA levels are expressed as log10 genome copies per gram of feces. Black arrows indicate HEV-3_p495 vaccination. Dotted lines indicate the detection limits.

Piglets were vaccinated intramuscularly with the HEV-3_p495 vaccine at 4, 6, and 8 weeks of age, beginning immediately after weaning (Figure 1A). Anti-HEV IgG became detectable in all vaccinated pigs by 6 weeks of age, 2 weeks after the first vaccination, and remained detectable in all animals through 21 weeks of age (Table 1). Antibody levels increased markedly after the second vaccination, reaching peak levels at 8 or 10 weeks of age, and subsequently declined gradually (Figure 2A). By 22–24 weeks of age, anti-HEV IgG had fallen below the cutoff in some vaccinated pigs (Table 1). The antibody response was comparable between pigs receiving 400 μg and those receiving 80 μg of HEV-3_p495 per dose. Notably, in pig No. I-1-1, anti-HEV IgG levels increased after 17 weeks of age, probably reflecting an anamnestic response to HEV exposure during cohabitation (Figure 2A).

At 12 weeks of age, the vaccinated and non-vaccinated pigs were exposed to HEV-3 through cohabitation with two naturally infected source pigs that were shedding HEV RNA in feces (Figure 1A). All three non-vaccinated control pigs subsequently became positive for fecal HEV RNA, with viral RNA first detected between 13 and 14 weeks of age; viremia was also detected during the course of infection (Figure 2B and Table 2). In contrast, HEV RNA was not detected in either fecal or serum samples from any of the pigs vaccinated with either 80 or 400 μg/dose throughout the observation period from 12 to 24 weeks of age (Figure 2B and Table 2). These results indicate that three-dose HEV-3_p495 vaccination at 4, 6, and 8 weeks of age protected juvenile pigs against detectable HEV-3 infection following natural exposure by cohabitation.

### 3.2. Protective Efficacy of Three- and Four-Dose HEV-3_p495 Vaccination Against Homologous Intravenous HEV-3 Challenge in Market-Age Pigs

**Figure 3:**
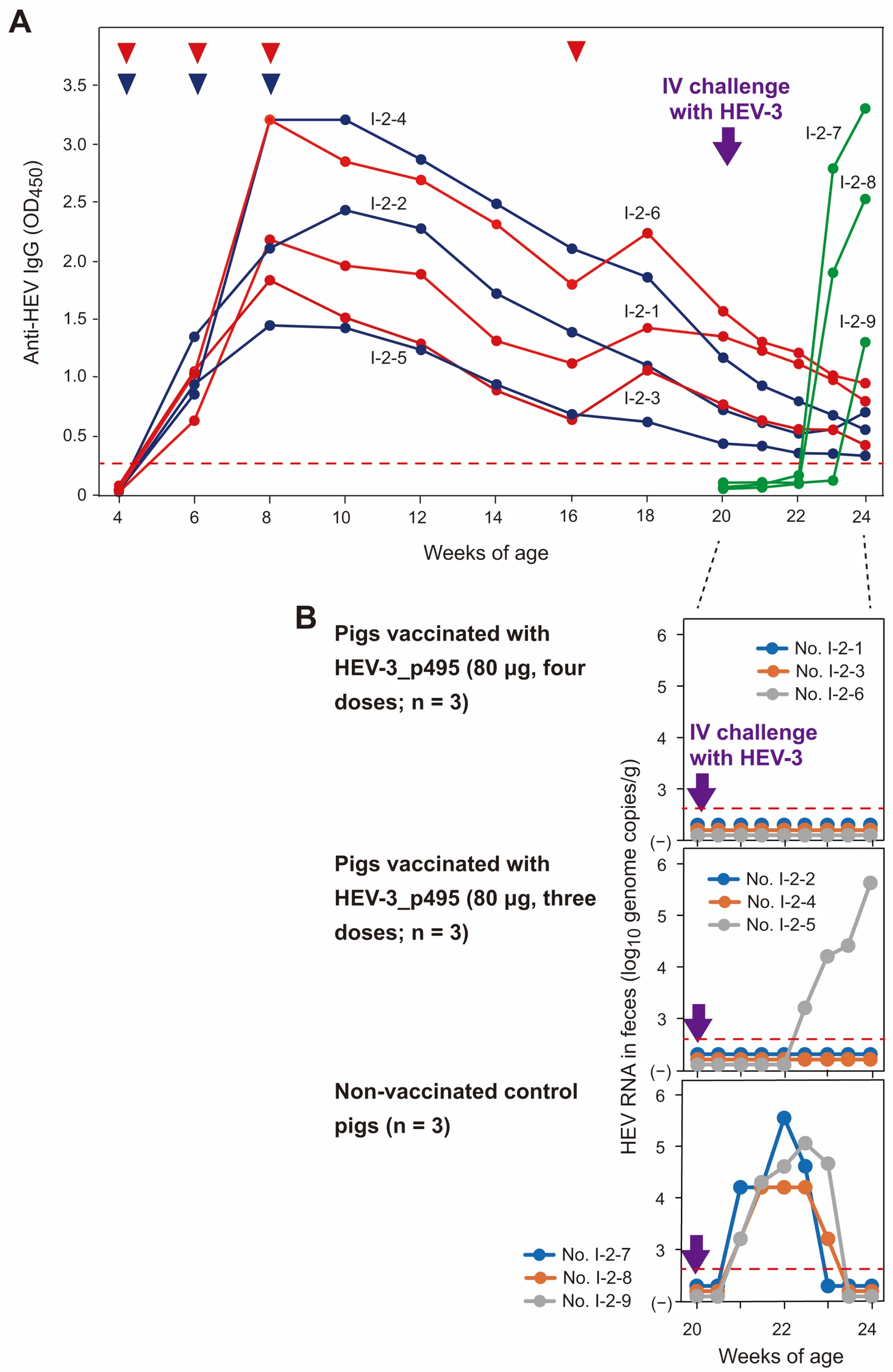
Anti-HEV IgG responses and fecal HEV RNA levels following homologous intravenous HEV-3 challenge in market-age pigs. (**A**) Anti-HEV IgG responses in pigs vaccinated with 80 μg of the HEV-3_p495 vaccine using a four-dose regimen (4, 6, 8, and 16 weeks of age; n = 3) or a three-dose regimen (4, 6, and 8 weeks of age; n = 3), and in non-vaccinated control pigs (n = 3). All pigs were intravenously challenged with HEV-3 at 20 weeks of age, as shown in Figure 1B. Anti-HEV IgG levels in serum are expressed as OD450 values. Red and blue lines represent pigs receiving the four-dose and three-dose regimens, respectively; vaccination time points for each regimen are indicated by arrows of the corresponding colors. Green lines represent non-vaccinated control pigs. (**B**) HEV RNA levels in feces from the vaccinated and non-vaccinated pigs following HEV-3 challenge. HEV RNA levels are expressed as log10 genome copies per gram of feces. The purple arrow indicates intravenous challenge with HEV-3. Dotted lines indicate the detection limits.

To evaluate protection against HEV-3 infection at market age, pigs were vaccinated with 80 μg of HEV-3_p495 at 4, 6, and 8 weeks of age (three-dose group) or at 4, 6, 8, and 16 weeks of age (four-dose group) and were intravenously challenged with HEV-3 at 20 weeks of age (Figure 1B). Anti-HEV IgG became detectable in all vaccinated pigs by 6 weeks of age and remained detectable through the end of the observation period at 24 weeks of age (Table 3). Antibody levels increased substantially after the second vaccination at 6 weeks of age but showed little or no further increase after the third vaccination at 8 weeks of age in most pigs (Figure 3A). In the four-dose group, administration of the booster dose at 16 weeks of age resulted in a renewed increase in anti-HEV IgG levels at 18 weeks, although the levels generally remained below the initial peak observed after the early vaccinations (Figure 3A).

Following intravenous HEV-3 challenge at 20 weeks of age, HEV RNA remained undetectable in both fecal and serum samples from all three pigs in the four-dose group throughout the 28-day observation period (Figure 3B and Table 4). In the three-dose group, two of the three pigs also remained negative for HEV RNA throughout the observation period. In contrast, pig No. I-2-5, which had the lowest anti-HEV IgG level among the vaccinated pigs at the time of challenge, became positive for fecal HEV RNA at 17 days post-inoculation and subsequently showed viremia (Figure 3 and Table 4). By comparison, all three non-vaccinated control pigs became positive for fecal HEV RNA by 7 days post-inoculation. Thus, the onset of detectable fecal HEV RNA in pig No. I-2-5 was delayed by 10 days relative to the non-vaccinated controls. Overall, the four-dose regimen prevented detectable HEV-3 infection in all vaccinated pigs, whereas the three-dose regimen prevented detectable infection in two of three pigs and delayed virus detection in the remaining pig. Consistent with these findings, HEV RNA was not detected in liver samples collected at necropsy at 24 weeks of age from the five vaccinated pigs that remained negative for fecal and serum HEV RNA, whereas liver HEV RNA was detected in pig No. I-2-5, which developed breakthrough infection.

### 3.3. Protective Efficacy of Two-Dose HEV-3_p495 Vaccination Against Heterologous HEV-4 Infection Following Cohabitation Challenge in Early-Age Pigs

**Figure 4:**
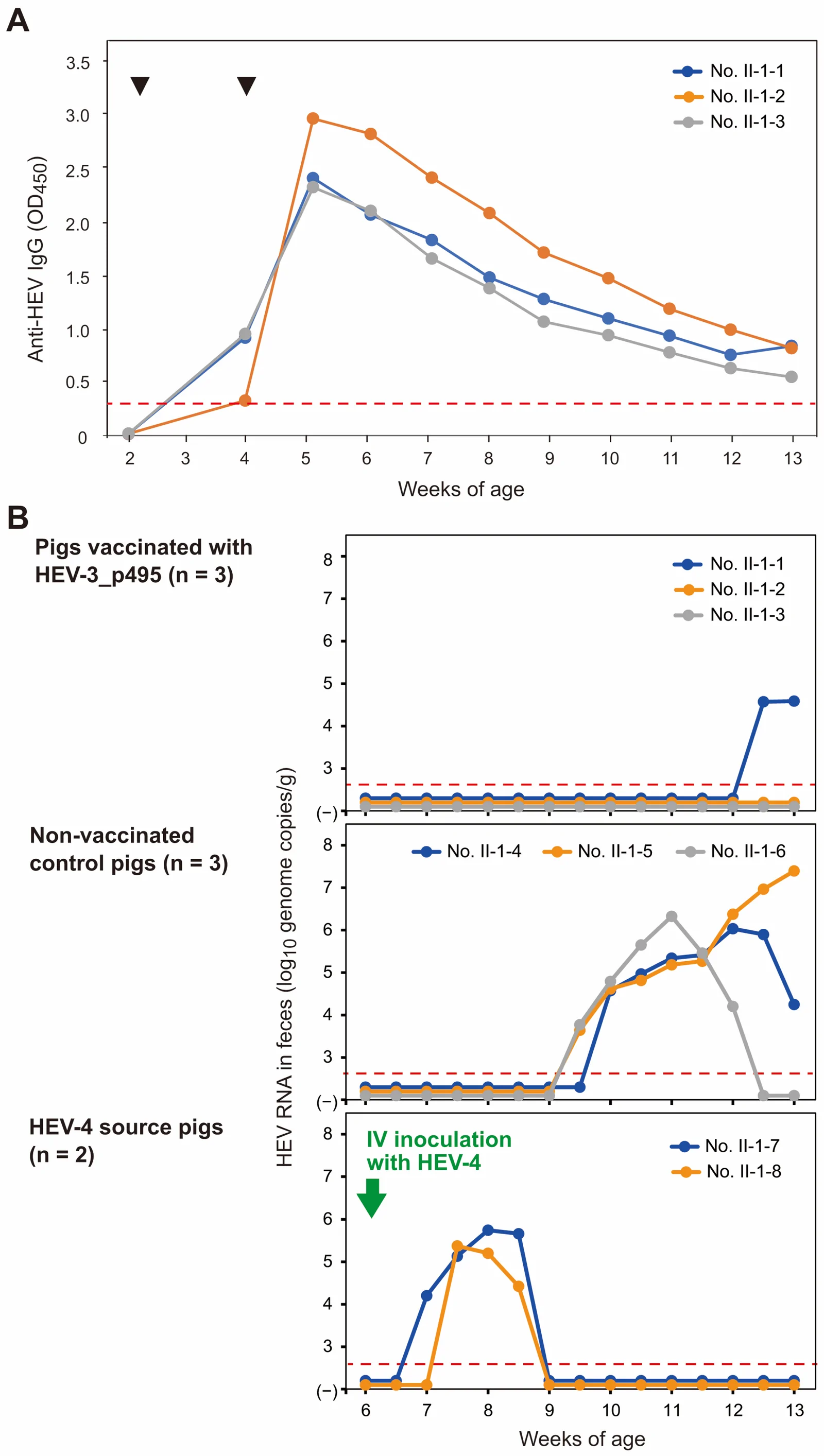
Anti-HEV IgG responses and fecal HEV RNA levels following heterologous HEV-4 cohabitation challenge in early-age pigs. (**A**) Anti-HEV IgG responses in pigs vaccinated with 80 μg of the HEV-3_p495 vaccine at 2 and 4 weeks of age (n = 3). Vaccinated pigs and non-vaccinated control pigs (n = 3) were co-housed with two HEV-4 source pigs. At 6 weeks of age, while cohabitation was continued, the source pigs were intravenously inoculated with HEV-4 to initiate the cohabitation challenge, as shown in Figure 1C. Anti-HEV IgG levels in serum are expressed as OD450 values. (**B**) HEV RNA levels in feces from the HEV-3_p495-vaccinated pigs, non-vaccinated control pigs, and HEV-4 source pigs. HEV RNA levels are expressed as log10 genome copies per gram of feces. Black arrows indicate HEV-3_p495 vaccination. The green arrow indicates intravenous inoculation of the source pigs with HEV-4. Dotted lines indicate the detection limits.

To evaluate cross-protection against heterologous HEV-4 infection early in life, piglets were vaccinated with 80 μg of HEV-3_p495 at 2 and 4 weeks of age and co-housed with two HEV-4 source pigs. At 6 weeks of age, the source pigs were intravenously inoculated with HEV-4 while cohabitation was continued (Figure 1C). Anti-HEV IgG was detectable in all three vaccinated pigs by 4 weeks of age and remained above the cutoff through the end of the observation period at 13 weeks of age (Figure 4A). Antibody levels peaked at 5 weeks of age, 1 week after the second vaccination, and subsequently declined gradually.

Following intravenous inoculation with HEV-4, both source pigs shed HEV RNA in feces, confirming exposure of the co-housed pigs to HEV-4 (Figure 4B and Table 5). All three non-vaccinated control pigs subsequently became positive for fecal HEV RNA, with the first positive samples detected between 9.5 and 10 weeks of age, and viremia was detected in all three animals. In contrast, two of the three HEV-3_p495-vaccinated pigs remained negative for HEV RNA in both fecal and serum samples throughout the observation period. In the remaining vaccinated pig (No. II-1-1), fecal HEV RNA was first detected at 12.5 weeks of age and remained detectable at 13 weeks, whereas serum HEV RNA was not detected. Notably, this pig showed a modest increase in anti-HEV IgG OD450 from 0.751 at 12 weeks to 0.833 at 13 weeks (Figure 4A). At necropsy at 13 weeks of age, HEV RNA was not detected in the livers of pigs No. II-1-2 and II-1-3, which remained negative for fecal and serum HEV RNA, whereas liver HEV RNA was detected in pig No. II-1-1, which showed delayed fecal virus shedding despite the absence of detectable viremia. Thus, two-dose HEV-3_p495 vaccination prevented detectable HEV-4 infection in two of three pigs and markedly delayed fecal virus detection in the remaining vaccinated pig compared with non-vaccinated controls.

### 3.4. HEV-3_p495 Vaccination Protects Pigs Against Heterologous HEV-4 Infection Following Cohabitation Challenge at Market Age

**Figure 5:**
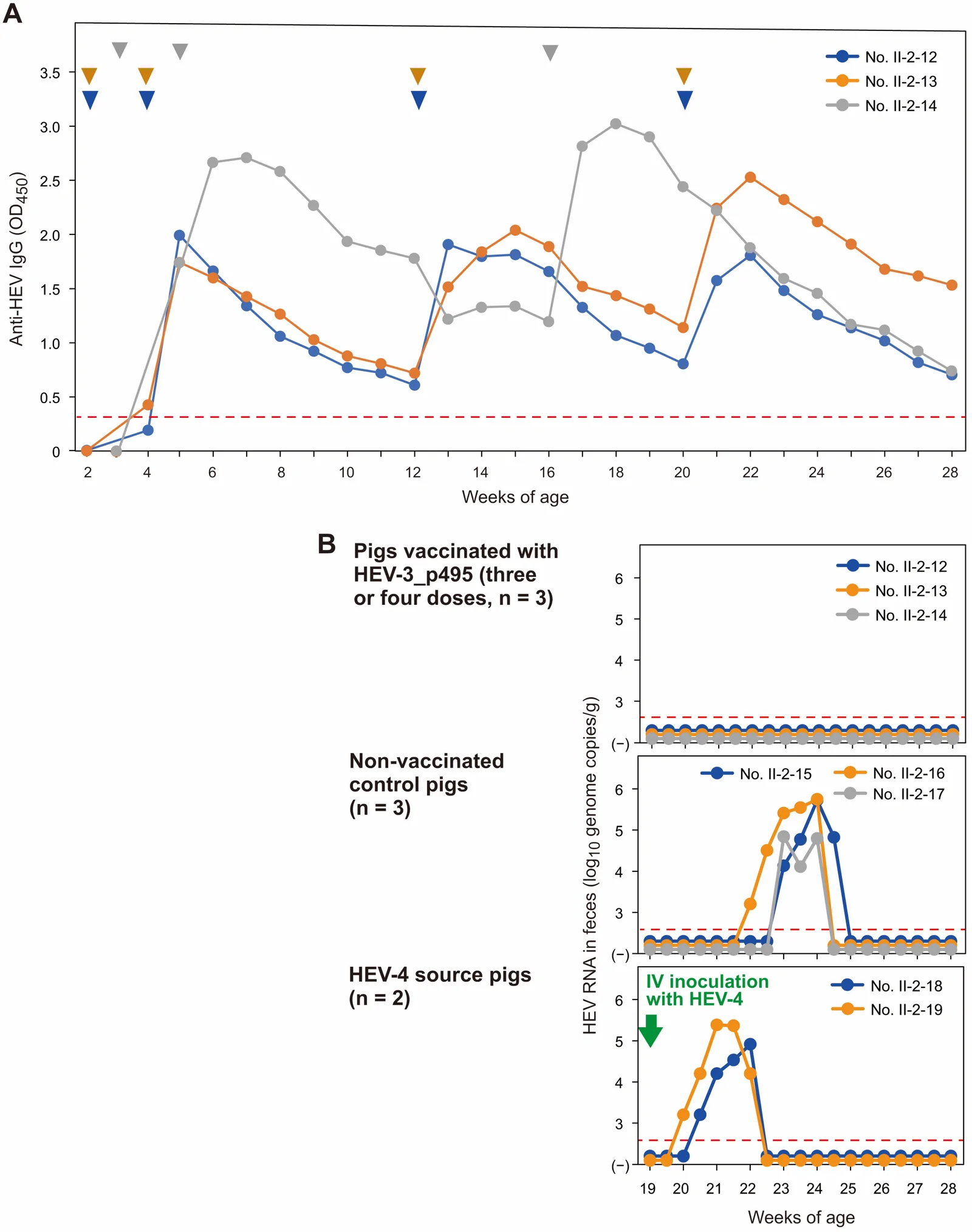
Anti-HEV IgG responses and fecal HEV RNA levels following heterologous HEV-4 cohabitation challenge in market-age pigs. (**A**) Anti-HEV IgG responses in three HEV-3_p495-vaccinated pigs (80 μg/dose), of which two pigs received the four-dose schedule at 2, 4, 12, and 20 weeks of age and one received the three-dose schedule at 3, 5, and 16 weeks of age. The two pigs receiving the four-dose schedule are shown by blue and orange lines, and the pig receiving the three-dose schedule is shown by a gray line; vaccination time points for each pig are indicated by arrows of the corresponding colors. These pigs were co-housed with two HEV-4 source pigs from 17 weeks of age. At 19 weeks of age, while cohabitation was continued, the source pigs were intravenously inoculated with HEV-4 to initiate the cohabitation challenge, as shown in Figure 1D. Anti-HEV IgG levels in serum are expressed as OD450 values. (**B**) HEV RNA levels in feces from the HEV-3_p495-vaccinated pigs, non-vaccinated control pigs, and HEV-4 source pigs. HEV RNA levels are expressed as log10 genome copies per gram of feces. The green arrow indicates intravenous inoculation of the source pigs with HEV-4. Dotted lines indicate the detection limits.

To further evaluate the cross-protective efficacy of the HEV-3_p495 vaccine against heterologous HEV-4 infection at market age, vaccinated and non-vaccinated pigs were subjected to a cohabitation challenge with HEV-4-infected source pigs (Figure 1D). Among the three vaccinated pigs included in the challenge experiment, two received four doses of HEV-3_p495 (80 μg/dose) at 2, 4, 12, and 20 weeks of age, whereas one received three doses at 3, 5, and 16 weeks of age. All three vaccinated pigs developed detectable anti-HEV IgG before initiation of the HEV-4 challenge, and antibody levels remained above the cutoff throughout the challenge period (Figure 5A).

The two source pigs were intravenously inoculated with HEV-4 at 19 weeks of age and subsequently shed HEV RNA in feces, confirming establishment of the transmission challenge (Figure 5B and Table 6). All three non-vaccinated control pigs subsequently became positive for fecal HEV RNA, and transient viremia was detected in two of the three animals (Table 6). In contrast, HEV RNA was not detected in either fecal or serum samples from any of the three HEV-3_p495-vaccinated pigs throughout the observation period up to 28 weeks of age (Figure 5B and Table 6). HEV RNA was also undetectable in liver samples collected from all three vaccinated pigs at necropsy at 28 weeks of age. These findings demonstrate that, under the conditions of this experiment, HEV-3_p495 vaccination provided complete protection against detectable heterologous HEV-4 infection following cohabitation exposure.

### 3.5. Anti-HEV IgG Responses Following Two Different HEV-3_p495 Vaccination Schedules

**Figure 6:**
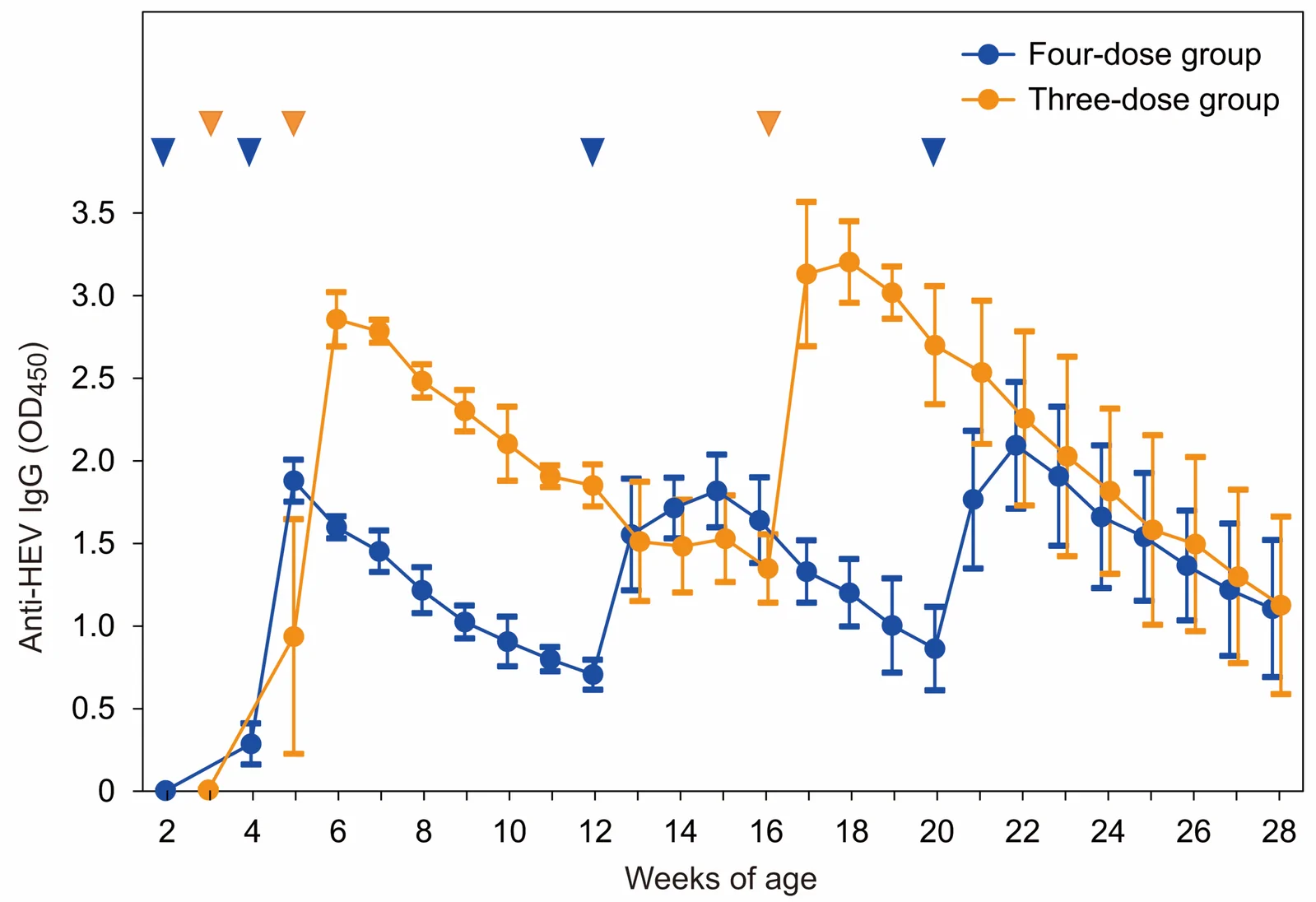
Anti-HEV IgG responses following three- and four-dose HEV-3_p495 vaccination schedules initiated at different ages. Anti-HEV IgG responses were descriptively compared between pigs receiving three doses (n = 3) and four doses (n = 3) of the HEV-3_p495 vaccine (80 μg/dose). The three-dose schedule was administered at 3, 5, and 16 weeks of age, whereas the four-dose schedule was administered at 2, 4, 12, and 20 weeks of age. The four-dose group is shown by a blue line, while the three-dose group is shown by an orange line; vaccination time points for each group are indicated by arrows of the corresponding colors. To obtain three animals per vaccination schedule, data from pigs included in the HEV-4 cohabitation challenge experiment (Figure 5) were combined with those from additional pigs that were vaccinated but not subjected to HEV challenge (Figure 1E). The four-dose group consisted of pigs No. II-2-12 and II-2-13 from the experiment shown in Figure 5 and an additional pig, No. II-2-23, shown in Figure 1E. The three-dose group consisted of pig No. II-2-14 from the experiment shown in Figure 5 and two additional pigs, Nos. II-2-21 and II-2-22, shown in Figure 1E. Pig No. II-2-21 died of pneumonia at 14 weeks of age; therefore, data from two pigs were available for the three-dose group from 15 weeks of age onward. Anti-HEV IgG levels in serum are expressed as OD450 values, and data are presented as the mean ± SD. Individual values are provided in Supplementary Table S2.

To compare the magnitude and duration of anti-HEV IgG response under different vaccination schedules, antibody levels were examined in pigs receiving three or four doses of HEV-3_p495 vaccine (80 μg/dose) (Figure 6 and Supplementary Table S2). The three-dose schedule consisted of vaccinations at 3, 5, and 16 weeks of age, whereas the four-dose schedule consisted of vaccinations at 2, 4, 12, and 20 weeks of age. From 6 through 12 weeks of age, mean anti-HEV IgG levels were higher in pigs receiving the three-dose schedule than in those receiving the four-dose schedule. In the three-dose group, the booster vaccination at 16 weeks of age was followed by a marked increase in anti-HEV IgG levels. In the four-dose group, antibody levels increased following the booster vaccinations at 12 and 20 weeks of age. Overall, these results demonstrate different temporal patterns of anti-HEV IgG responses under the two vaccination schedules examined. Because the schedules differed in the age at primary vaccination, vaccination intervals, booster timing, and total number of doses, these differences cannot be attributed specifically to the number of vaccine doses. In addition, because some pigs included in this comparison were subsequently exposed to HEV-4 in the cohabitation challenge experiment, antibody responses after initiation of viral exposure should be interpreted cautiously.

## 4. Discussion

The present study demonstrates that immunization of pigs with the *E. coli*-produced HEV-3 VLP vaccine HEV-3_p495 confers robust protection against both homologous HEV-3 and heterologous HEV-4 infection. Three findings are particularly important. First, three-dose vaccination completely prevented detectable HEV-3 infection in juvenile pigs during prolonged cohabitation with naturally infected source pigs, and comparable protection was achieved with 80 and 400 μg of HEV-3_p495 per dose. Second, protection was maintained to market age, with complete protection against intravenous HEV-3 challenge in pigs receiving a late booster and substantial protection even in pigs receiving only the three-dose primary regimen. Third, and most notably, an HEV-3-derived VLP conferred cross-protection against heterologous HEV-4 following cohabitation challenge in both early-age and market-age pigs. Thus, HEV-3_p495 induced protective immunity across different ages, exposure routes, and viral genotypes, supporting further evaluation of this VLP as a candidate swine vaccine for reducing HEV infection and shedding.

The protection observed against homologous HEV-3 is noteworthy in the context of previous vaccination studies in pigs. Earlier studies using recombinant HEV capsid antigens have generally shown variable protection, ranging from reductions in viremia or fecal shedding to incomplete prevention of infection. Sanford et al. reported that immunization of pigs with truncated recombinant capsid antigens induced strong anti-HEV IgG responses but provided only partial protection against HEV-3 challenge [29]. More recently, Dähnert et al. showed that both the licensed genotype 1-derived Hecolin^®^ vaccine and a genotype 3-derived p239 antigen induced high neutralizing antibody titers in pigs but did not completely prevent HEV-3 infection; viral RNA remained detectable in the liver and/or bile of most vaccinated animals [32]. In contrast, Go et al. demonstrated dose-dependent protection with a baculovirus-produced HEV-3-239-VLP vaccine, in which pigs receiving 200 μg were negative for HEV RNA in serum and feces following challenge, whereas transient viremia and fecal shedding occurred in pigs receiving 100 μg [30]. Against this background, the absence of detectable HEV RNA in both serum and feces of all pigs receiving three doses of either 80 or 400 μg of HEV-3_p495 during the 12-week observation period after initiation of cohabitation is particularly encouraging. All non-vaccinated controls became infected under the same conditions, confirming effective viral exposure. Moreover, antibody responses were broadly comparable between the 80- and 400-μg groups, suggesting that increasing the antigen dose fivefold did not confer an obvious immunological or virological advantage under the conditions examined. These findings support 80 μg per dose as a reasonable dose for further evaluation and optimization.

The market-age HEV-3 challenge further suggests that the durability of vaccine-induced immunity may be important for maintaining protection until slaughter age. Anti-HEV IgG remained detectable in vaccinated pigs through 24 weeks of age (Figure 3A, Table 3), and administration of a fourth dose at 16 weeks produced a renewed increase in antibody levels before challenge (Figure 3A). Following intravenous challenge at 20 weeks of age, all three pigs in the four-dose group remained negative for HEV RNA in serum and feces, whereas two of three pigs in the three-dose group were similarly protected. The only breakthrough infection occurred in pig No. I-2-5, which had the lowest anti-HEV IgG level among vaccinated animals at the time of challenge. Importantly, fecal HEV RNA became detectable 17 days after inoculation, 10 days later than in the non-vaccinated controls, indicating that vaccination substantially delayed infection even in this animal (Figure 3B, Table 4). Although these observations are compatible with an association between the magnitude of antibody responses at exposure and protection, they should not be interpreted as defining a protective antibody threshold. The ELISA used here measures total anti-HEV IgG rather than virus-neutralizing activity, and the number of animals was too small for formal correlation analysis. Nevertheless, these findings raise the possibility that booster immunization timed to maintain antibody responses through the late production period could improve the consistency of protection.

A major contribution of the present study is the demonstration of cross-protection against heterologous HEV-4. Although mammalian HEV strains have historically been considered to constitute a single serotype, studies have identified both broadly cross-reactive conformational neutralizing epitopes and genotype-associated neutralizing epitopes within ORF2 [40,41,42,43]. Antibodies raised against recombinant ORF2 regions can cross-neutralize multiple HEV genotypes, and structural analysis has identified conserved residues within the protruding capsid domain that contribute to cross-genotype antibody recognition [40,41,42]. Notably, Wen et al. identified both broadly reactive neutralizing antibodies and antibodies with genotype-associated specificity, including an antibody capable of recognizing and neutralizing HEV-3 and HEV-4 [43]. These observations indicate that cross-genotype immunity may reflect recognition of both shared and genotype-associated antigenic determinants. Consistent with this concept of cross-protective immunity, prior infection of pigs with swine HEV-3 has been shown to confer protection against subsequent challenge with both HEV-3 and HEV-4 [29]. However, direct experimental evidence that an HEV-3-based vaccine can protect pigs against HEV-4 has remained limited. The present findings therefore provide important evidence that immunity elicited by HEV-3_p495 can recognize protective antigenic determinants shared between HEV-3 and HEV-4.

Cross-protection was already apparent after only two doses administered early in life. Following HEV-4 exposure by cohabitation, two of three vaccinated pigs remained negative for HEV RNA in both serum and feces, whereas all three non-vaccinated control pigs became infected. In the remaining vaccinated animal, No. II-1-1, fecal HEV RNA was first detected substantially later than in controls and viremia was not detected. These findings indicate that the two-dose regimen induced biologically meaningful cross-protection, although protection was not complete in every animal. This distinction is important because it suggests that primary vaccination alone may suppress or delay heterologous infection, whereas sustained or boosted immunity may be required for more consistent protection later in life.

Indeed, the protection observed in the market-age HEV-4 cohabitation experiment was particularly strong. Following inoculation of the HEV-4 source pigs, both source animals shed viral RNA, and all three non-vaccinated control pigs became positive for fecal HEV RNA; transient viremia was also detected in two animals. In contrast, HEV RNA was not detected in either serum or feces from any of the vaccinated pigs throughout the observation period up to 28 weeks of age (Figure 5B, Table 6). The occurrence of infection in all control pigs provides an important internal validation of the challenge model and indicates that the absence of detectable infection among vaccinated animals was not attributable to insufficient exposure. Taken together with the early-age experiment, these results suggest that HEV-3_p495 elicits cross-reactive immunity capable of protecting against HEV-4 and that the level and timing of immunity at exposure may influence the completeness of protection. The precise immunological mechanism underlying this cross-protection remains to be established. Although previous studies have demonstrated cross-genotype neutralizing epitopes within ORF2, the present study did not directly assess neutralizing activity or other immune mechanisms. Therefore, whether the observed HEV-3/HEV-4 cross-protection is mediated by cross-neutralizing antibodies, cellular immunity, mucosal immunity, or a combination of these responses cannot be determined from the present data.

The use of cohabitation challenge models is another important feature of this study. Experimental HEV vaccine studies frequently use intravenous inoculation because it provides a controlled and reproducible infectious dose. In the present study, preliminary experiments confirmed that intravenous inoculation with 10^3^, 10^4^, or 10^5^ genome copies of the HEV-3 swEJM2100729F strain established infection (Supplementary Table S1), supporting the use of 10^4^ copies for the market-age challenge. Nevertheless, intravenous inoculation bypasses the natural route of HEV transmission. In commercial swine populations, pigs are primarily exposed through the fecal–oral route and may experience repeated environmental exposure over extended periods. HEV transmission occurs predominantly through fecal-oral exposure, with both direct contact and contamination of the environment contributing to transmission [34,44,45]. Pigs may therefore experience repeated exposure over extended periods rather than a single defined infectious dose. The cohabitation experiments used here therefore provide a complementary and more biologically relevant assessment of vaccine performance. Juvenile pigs were continuously exposed to naturally HEV-3-infected source pigs from 12 to 20 weeks of age and were subsequently monitored until 24 weeks of age, whereas the HEV-4 experiments similarly relied on infected source pigs to generate ongoing exposure. The consistent infection of non-vaccinated control animals demonstrates that these models generated effective transmission pressure. Protection under such conditions strengthens the translational relevance of HEV-3_p495 compared with protection demonstrated solely after a single artificial inoculation.

The antibody kinetics observed under the two vaccination schedules may provide useful information for the design of future regimen-optimization studies. The three-dose schedule administered at 3, 5, and 16 weeks of age and the four-dose schedule administered at 2, 4, 12, and 20 weeks of age produced different temporal patterns of anti-HEV IgG responses (Figure 6, Supplementary Table S2). In particular, antibody levels increased markedly following vaccination at 16 weeks in the three-dose group and following vaccination at 20 weeks in the four-dose group. However, these observations should be interpreted cautiously because the two schedules differed simultaneously in the age at primary vaccination, vaccination intervals, booster timing, and total number of doses. In addition, one pig in the three-dose group died of pneumonia at 14 weeks of age, leaving only two animals for later observation, and some pigs included in this comparison were subsequently exposed to HEV-4 in the cohabitation challenge experiment. Therefore, the present data do not permit conclusions regarding the relative contribution of vaccination timing, interval, or dose number. A dedicated regimen-optimization study using larger groups and appropriately matched vaccination schedules will be required to determine the optimal number and timing of various doses.

The present findings may have particular relevance from a One Health perspective. HEV-3 and HEV-4 are zoonotic genotypes for which domestic pigs constitute a major animal reservoir, and consumption of raw or undercooked pork and pork products represents an established route of human exposure. Control of HEV infection in pigs may therefore represent a potential means of reducing foodborne transmission to humans. A vaccine that prevents infection or markedly suppresses fecal shedding could theoretically reduce both within-herd transmission and environmental contamination and, consequently, decrease the proportion of HEV-infected pigs entering the food chain. Transmission modeling supports the potential of vaccination to reduce HEV circulation and the number of infectious pigs at slaughter, while farm-level studies indicate that measures limiting HEV transmission during the finishing period may reduce infection at the end of production [22,27]. The demonstration of protection against both HEV-3 and HEV-4 is advantageous because these zoonotic genotypes have overlapping but geographically distinct distributions. Importantly, however, the present experiments assessed individual-level protection under controlled conditions and did not directly evaluate herd-level transmission, viral prevalence at slaughter, or contamination of edible tissues. The broader public-health impact of swine vaccination therefore remains to be demonstrated experimentally.

Several limitations should be considered when interpreting these findings. First, the number of pigs in each experimental group was small, generally three animals, which limits statistical power and results in considerable uncertainty in estimating vaccine efficacy and differences among vaccination regimens. Accordingly, the present study should be regarded as a laboratory-scale proof-of-concept study demonstrating the potential protective efficacy of HEV-3_p495 under controlled experimental conditions rather than as a definitive evaluation of vaccine efficacy under field conditions. Larger studies involving commercial swine populations will be required to confirm the reproducibility and generalizability of these findings before practical field application can be considered. Second, age at vaccination, number and timing of doses, challenge age, challenge route, and viral genotype differed among experiments. In particular, the three- and four-dose schedules were not designed as a controlled comparison of dose number because they also differed in the age at primary vaccination, vaccination intervals, and booster timing. Moreover, one pig in the three-dose group died of pneumonia, further limiting comparison between the schedules. Therefore, the present data do not establish the optimal number or timing of vaccine doses. Third, immune responses were evaluated primarily using serum anti-HEV IgG ELISA; neutralizing antibody activity, antibody avidity, HEV-specific cellular immune responses, and intestinal mucosal IgA were not directly assessed. Mucosal immunity may be particularly relevant to protection against fecal–oral transmission in the cohabitation model. Consequently, the present study demonstrates protective efficacy but does not define the immune mechanisms or correlates responsible for protection. Fourth, the present experiments evaluated protection at the individual-animal level and did not directly assess herd-level transmission, HEV prevalence at slaughter, or contamination of edible tissues. Thus, whether vaccination can reduce viral circulation within commercial swine herds and ultimately decrease zoonotic exposure remains to be determined in larger field studies. Finally, although HEV RNA was not detected in the liver at necropsy in vaccinated pigs that remained negative for fecal and serum HEV RNA, liver samples were unavailable from vaccinated pigs in the juvenile-stage HEV-3 cohabitation experiment, and infection below the detection limit cannot be completely excluded.

## 5. Conclusions

In conclusion, immunization with the *E. coli*-produced HEV-3_p495 VLP induced robust protective immunity in pigs against homologous HEV-3 and, importantly, heterologous HEV-4 challenge. Protection was demonstrated not only after controlled intravenous challenge but also in cohabitation models that reproduce key features of natural fecal–oral transmission, with complete prevention of detectable viremia and fecal shedding in several experimental settings. The findings further suggest that maintaining vaccine-induced immunity through appropriately timed booster vaccination may be important for consistent protection at market age. Although larger studies are required to define immune correlates, optimal vaccination schedules, breadth of cross-protection, and effectiveness under field conditions, HEV-3_p495 represents a promising swine vaccine candidate. The present proof-of-concept findings provide a rationale for future herd-level studies to determine whether vaccination can reduce HEV transmission within swine populations and decrease the prevalence of infected pigs at slaughter.

## Figures and Tables

**Table 1:** The number of pigs with anti-HEV IgG antibodies detected in serum samples collected at the indicated ages in the juvenile-stage experiment (cohabitation challenge at 12 weeks of age).

Group	Weeks of Age															
	4 (1st vac)	6 (2nd vac)	8 (3rd vac)	10	12 ^a^	14	15	16	17	18	19	20	21	22	23	24
HEV-3_p495 (400 µg/dose) ^b^	0/3	3/3	3/3	3/3	3/3	3/3	3/3	3/3	3/3	3/3	3/3	3/3	3/3	2/3	2/3	2/3
HEV-3_p495 (80 µg/dose) ^c^	0/3	3/3	3/3	3/3	3/3	3/3	3/3	3/3	3/3	3/3	3/3	3/3	3/3	3/3	3/3	2/3
Non-vaccinated ^d^	– ^e^	–	–	–	0/3	0/3	2/3	3/3	3/3	3/3	3/3	3/3	3/3	3/3	3/3	3/3

vac, vaccination. ^a^ Cohabitation with HEV-3-infected pigs was initiated. ^b^ Pig No. (I-1-1, 3, and 5). ^c^ Pig No. (I-1-2, 4, and 6). ^d^ Pig No. (I-1-7 to I-1-9). ^e^ Not tested.

**Table 2:** The number of pigs with HEV RNA detected in fecal and serum samples collected on the indicated days in the juvenile-stage experiment (HEV-3 cohabitation challenge at 12 weeks of age).

Group	Days After the Initiation of Cohabitation Challenge (Weeks of Age)													
	0 (12)	3	7 (13)	10	14 (14)	17	21 (15)	24	28 (16)	35 (17)	42 (18)	56 (20)	70 (22)	84 (24)
HEV-3_p495 (400 µg/ dose) ^a^	0/3 • 0/3	0/3	0/3	0/3	0/3 • 0/3	0/3	0/3 • 0/3	0/3	0/3 • 0/3	0/3 • 0/3	0/3 • 0/3	0/3 • 0/3	0/3 • 0/3	0/3 • 0/3
HEV-3_p495 (80 µg/ dose) ^b^	0/3 • 0/3	0/3	0/3	0/3	0/3 • 0/3	0/3	0/3 • 0/3	0/3	0/3 • 0/3	0/3 • 0/3	0/3 • 0/3	0/3 • 0/3	0/3 • 0/3	0/3 • 0/3
Non-vaccinated ^c^	0/3 • 0/3	0/3	1/3	2/3	3/3 • 2/3	3/3	3/3 • 3/3	2/3	0/3 • 0/3	0/3 • 0/3	0/3 • 0/3	0/3 • 0/3	0/3 • 0/3	0/3 • 0/3
Infection source ^d^	2/2	2/2	2/2	2/2	2/2	1/2	1/2	0/2	0/2	0/2	0/2	0/2	‡	‡

‡, not tested. The numbers of pigs positive for HEV RNA in fecal and serum samples are shown above and below the dots (•), respectively. ^a^ Pig No. (I-1-1, 3, and 5). ^b^ Pig No. (I-1-2, 4, and 6). ^c^ Pig No. (I-1-7 to I-1-9). ^d^ Pig No. (I-1-10 and I-1-11); serum samples were not collected from the two pigs, which were removed at 20 weeks of age.

**Table 3:** The number of pigs with anti-HEV IgG antibodies detected in serum samples collected at the indicated ages in the market-age experiment (intravenous HEV-3 challenge at 20 weeks of age).

Group	Weeks of Age												
	4 (1st vac)	6 (2nd vac)	8 (3rd vac)	10	12	14	16 (4th vac)	18	20 ^a^	21	22	23	24
HEV-3_p495 (80 µg/dose), four doses ^b^	0/3	3/3	3/3	3/3	3/3	3/3	3/3	3/3	3/3	3/3	3/3	3/3	3/3
HEV-3_p495 (80 µg/dose), three doses ^c^	0/3	3/3	3/3	3/3	3/3	3/3	3/3	3/3	3/3	3/3	3/3	3/3	3/3

vac, vaccination. ^a^ HEV-3 (10^4^ genome copies/pig) was administered intravenously. ^b^ Pig No. (I-2-1, 3, and 6). ^c^ Pig No. (I-2-2, 4, and 5).

**Table 4:** Detection of HEV RNA in fecal and serum samples following intravenous HEV-3 challenge at 20 weeks of age.

Group	Detection of HEV RNA in Fecal/Serum Samples									
	Pig No.	Days After Inoculation of HEV-3 (Weeks of Age)								
		0 (20)	3	7 (21)	10	14 (22)	17	21 (23)	24	28 (24)
HEV-3_p495 (80 µg/dose), four doses	I-2-1	−/−	−/‡	−/−	−/‡	−/−	−/‡	−/−	−/‡	−/−
	I-2-3	−/−	−/‡	−/−	−/‡	−/−	−/‡	−/−	−/‡	−/−
	I-2-6	−/−	−/‡	−/−	−/‡	−/−	−/‡	−/−	−/‡	−/−
HEV-3_p495 (80 µg/dose), three doses	I-2-2	−/−	−/‡	−/−	−/‡	−/−	−/‡	−/−	−/‡	−/−
	I-2-4	−/−	−/‡	−/−	−/‡	−/−	−/‡	−/−	−/‡	−/−
	I-2-5	−/−	−/‡	−/−	−/‡	−/−	+/‡	+/+	+/‡	+/+
Non-vaccinated	I-2-7	−/−	−/‡	+/−	+/‡	+/+	+/‡	−/−	−/‡	−/−
	I-2-8	−/−	−/‡	+/−	+/‡	+/+	+/‡	+/−	−/‡	−/−
	I-2-9	−/−	−/‡	+/−	+/‡	+/+	+/‡	+/−	−/‡	−/−

Fecal and serum sample data are presented before and after the slash, respectively. +, positive; −, negative; ‡, not tested. At necropsy at 24 weeks of age, liver samples from vaccinated pigs I-2-1, I-2-2, I-2-3, I-2-4, and I-2-6 were negative for HEV RNA, whereas that from pig I-2-5 was positive.

**Table 5:** Detection of HEV RNA in fecal and serum samples following initiation of HEV-4 exposure by intravenous inoculation of source pigs at 6 weeks of age.

Group	Detection of HEV RNA in Fecal/Serum Samples															
	Pig No.	Days After Intravenous HEV-4 Inoculation of Source Pigs (Weeks of Age)														
		0 (6)	3	7 (7)	10	14 (8)	17	21 (9)	24	28 (10)	31	35 (11)	38	42 (12)	45	49 (13)
HEV-3_p495 (80 µg/dose), two doses	II-1-1	−/−	−/ ‡	−/−	−/ ‡	−/−	−/ ‡	−/−	−/ ‡	−/−	−/ ‡	−/−	−/ ‡	−/−	+/ ‡	+/−
	II-1-2	−/−	−/ ‡	−/−	−/ ‡	−/−	−/ ‡	−/−	−/ ‡	−/−	−/ ‡	−/−	−/ ‡	−/−	−/ ‡	−/−
	II-1-3	−/−	−/ ‡	−/−	−/ ‡	−/−	−/ ‡	−/−	−/ ‡	−/−	−/ ‡	−/−	−/ ‡	−/−	−/ ‡	−/−
Non-vaccinated	II-1-4	−/−	−/ ‡	−/−	−/ ‡	−/−	−/ ‡	−/−	−/ ‡	+/−	+/ ‡	+/+	+/ ‡	+/+	+/ ‡	+/−
	II-1-5	−/−	−/ ‡	−/−	−/ ‡	−/−	−/ ‡	−/−	+/ ‡	+/−	+/ ‡	+/+	+/ ‡	+/+	+/ ‡	+/+
	II-1-6	−/−	−/ ‡	−/−	−/ ‡	−/−	−/ ‡	−/−	+/ ‡	+/+	+/ ‡	+/+	+/ ‡	+/−	−/ ‡	−/−
HEV-4 source pigs	II-1-7	−/−	−/ ‡	+/−	+/ ‡	+/+	+/ ‡	−/−	−/ ‡	−/−	−/ ‡	−/−	−/ ‡	−/−	−/ ‡	−/−
	II-1-8	−/−	−/ ‡	−/+	+/ ‡	+/+	+/ ‡	−/−	−/ ‡	−/−	−/ ‡	−/−	−/ ‡	−/−	−/ ‡	−/−

Fecal and serum sample data are presented before and after the slash, respectively. +, positive; −, negative; ‡, not tested. At necropsy at 13 weeks of age, liver samples from vaccinated pigs II-1-2 and II-1-3 were negative for HEV RNA, whereas that from pig II-1-1 was positive.

**Table 6:** Detection of HEV RNA in fecal and serum samples following initiation of HEV-4 exposure by intravenous inoculation of source pigs at 19 weeks of age.

Group	Detection of HEV RNA in Fecal/Serum Samples																			
	Pig No.	Days After Intravenous HEV-4 Inoculation of Source Pigs/Weeks of Age																		
		0 / 19	3	7 / 20	10	14 / 21	17	21 / 22	24	28 / 23	31	35 / 24	38	42 / 25	45	49 / 26	52	56 / 27	59	63 / 28
HEV-3_ p495 (80 µg/dose), three or four doses ^a^	II- 2-12	−/−	−/ ‡	−/−	−/ ‡	−/−	−/ ‡	−/−	−/ ‡	−/−	−/ ‡	−/−	−/ ‡	−/−	−/ ‡	−/−	−/ ‡	−/−	−/ ‡	−/−
	II- 2-13	−/−	−/ ‡	−/−	−/ ‡	−/−	−/ ‡	−/−	−/ ‡	−/−	−/ ‡	−/−	−/ ‡	−/−	−/ ‡	−/−	−/ ‡	−/−	−/ ‡	−/−
	II- 2-14	−/−	−/ ‡	−/−	−/ ‡	−/−	−/ ‡	−/−	−/ ‡	−/−	−/ ‡	−/−	−/ ‡	−/−	−/ ‡	−/−	−/ ‡	−/−	−/ ‡	−/−
Non-vaccinated	II- 2-15	−/−	−/ ‡	−/−	−/ ‡	−/−	−/ ‡	−/−	−/ ‡	+/−	+/ ‡	+/+	+/ ‡	−/−	−/ ‡	−/−	−/ ‡	−/−	−/ ‡	−/−
	II- 2-16	−/−	−/ ‡	−/−	−/ ‡	−/−	−/ ‡	+/−	+/ ‡	+/+	+/ ‡	+/+	−/ ‡	−/−	−/ ‡	−/−	−/ ‡	−/−	−/ ‡	−/−
	II- 2-17	−/−	−/ ‡	−/−	−/ ‡	−/−	−/ ‡	−/−	−/ ‡	+/−	+/ ‡	+/−	−/ ‡	−/−	−/ ‡	−/−	−/ ‡	−/−	−/ ‡	−/−
HEV-4 source pigs	II- 2-18	−/−	−/ ‡	−/−	−/ ‡	+/+	+/ ‡	+/−	−/ ‡	−/−	−/ ‡	−/−	−/ ‡	−/−	−/ ‡	−/−	−/ ‡	−/−	−/ ‡	−/−
	II- 2-19	−/−	−/ ‡	+/−	−/ ‡	+/−	+/ ‡	+/−	−/ ‡	−/−	−/ ‡	−/−	−/ ‡	−/−	−/ ‡	−/−	−/ ‡	−/−	−/ ‡	−/−

Fecal and serum sample data are presented before and after the slash, respectively. +, positive; −, negative; ‡, not tested. At necropsy at 28 weeks of age, liver samples from all three vaccinated pigs (II-2-12, II-2-13, and II-2-14) were negative for HEV RNA. ^a^ Pigs II-2-12 and II-2-13 received four doses, whereas pig II-2-14 received three doses.

## Data Availability

All data generated or analyzed in this study are included in the manuscript and its Supplementary Materials.

## Supplementary Materials

The following supporting information can be downloaded at https://www.mdpi.com/article/10.3390/v18101122/s1, Table S1: Virological and Serological Responses of Pigs Intravenously Inoculated with the Different Doses of the HEV-3 swEJM2100729F Strain; Table S2: Anti-HEV IgG Responses in Pigs Receiving Four- and Three-Dose HEV-3_p495 Vaccination Schedules.

## Abbreviations

The following abbreviations are used in this manuscript:

HEV	Hepatitis E virus
WHO	World Health Organization
FAO	Food and Agriculture Organization
HEV-1–HEV-8	HEV genotypes 1–8
ORF	Open reading frame
HEV239	Recombinant 239-amino acid HEV-1 ORF2 protein
*E. coli*	*Escherichia coli*
VLPs	Virus-like particles
HEV-3_p495	HEV-3 VLP vaccine
PBS	Phosphate-buffered saline
0.2% BSA-PBS	PBS containing 0.2% bovine serum albumin
ELISA	Enzyme-linked immunosorbent assay
OD	Optical density
PCR	Polymerase chain reaction
bp	Base pairs
Real-time RT-PCR	Real-time reverse transcription PCR
SD	Standard deviation
