# Supplementary material for: Immunization with an Escherichia coli-Produced Hepatitis E Virus Genotype 3 Virus-like Particle (HEV-3_p495) Confers Protection Against HEV-3 and HEV-4 Challenge in Pigs
Source: Viruses. 2026 Oct 10;18(10):1122. doi: 10.3390/v18101122 (PMC13709936; doi:10.3390/v18101122)
Supplement: Supplementary file 1 [file viruses-18-01122-s001.zip › Table S1.pdf]

Table S1.

Virological and Serological Responses of Pigs Intravenously Inoculated with the Different Doses of the HEV-3 swEJM2100729F Strain.

	Pig No.	Inoculum doses (genome copies, i.v.)	Day 0	Day 2	Day 6	Day 9	Day 13	Day 16	Day 20	Day 23	Day 27	Day 30
Anti-HEV IgG (OD450)	1218	10^3	0.061 -		0.057 -		0.060 -		0.309 +		1.799 +	1.814 +
	1219		0.066 -		0.066 -		0.078 -		0.804 +		2.667 +	2.581 +
	1220	10^4	0.058 -		0.062 -		0.058 -		1.623 +		2.498 +	2.597 +
	1221		0.034 -		0.036 -		0.048 -		1.731 +		2.696 +	2.669 +
	1233	10^5	0.135 -		0.131 -		0.124 -		1.958 +		2.728 +	2.776 +
	1245		0.062 -		0.065 -		0.062 -		2.470 +		3.123 +	3.103 +
HEV RNA in feces (genome copies/g)	1218	10^3	-	-	-	1.6E+03	1.6E+04	4.2E+04	1.6E+03	-	-	-
	1219		-	-	-	1.6E+04	1.6E+04	2.2E+05	1.6E+04	-	-	-
	1220	10^4	-	-	1.6E+04	1.6E+04	1.9E+05	1.6E+04	-	-	-	-
	1221		-	-	1.6E+04	2.0E+04	5.2E+04	1.6E+04	-	-	-	-
	1233	10^5	-	-	1.6E+03	1.1E+05	4.6E+05	4.7E+04	-	-	-	-
	1245		-	-	1.6E+04	2.1E+05	6.5E+05	4.9E+04	-	-	-	-
HEV RNA in serum	1218	10^3	-		-		+		-		-	-
	1219		-		-		+		-		-	-
	1220	10^4	-		+		+		-		-	-
	1221		-		+		-		-		-	-
	1233	10^5	-		+		+		-		-	-
	1245		-		+		+		-		-	-

+, positive; -, negative; gray cells, not tested. For anti-HEV IgG, samples with OD450 values ≥0.274 were considered positive. HEV RNA levels in feces are expressed as genome copies/g.
