# Supplementary material for: Immunization with an Escherichia coli-Produced Hepatitis E Virus Genotype 3 Virus-like Particle (HEV-3_p495) Confers Protection Against HEV-3 and HEV-4 Challenge in Pigs
Source: Viruses. 2026 Oct 10;18(10):1122. doi: 10.3390/v18101122 (PMC13709936; doi:10.3390/v18101122)
Supplement: Supplementary file 1 [file viruses-18-01122-s001.zip › Table S2.pdf]

Table S2.

Anti-HEV IgG Responses in Pigs Receiving Four- and Three-Dose HEV-3_p495 Vaccination Schedules.

Weeks of age	Four-dose vaccination group					Three-dose vaccination group				
	Point	Anti-HEV IgG (OD450)				Point	Anti-HEV IgG (OD450)			
		Pig No. II-2-12	Pig No. II-2-13	Pig No. II-2-23	Mean ± SD		Pig No. II-2-14	Pig No. II-2-21	Pig No. II-2-22	Mean ± SD
2	Pre-imm.	0.006	0.002	0.004	0.004 ± 0.002					
3						Pre-imm.	0.006	0.013	0.009	0.009 ± 0.004
4	2W/1st	0.192	0.427	0.239	0.286 ± 0.124					
5	1W/2nd	1.993	1.742	1.904	1.880 ± 0.127	2W/1st	1.744	0.655	0.409	0.936 ± 0.710
6	2W/2nd	1.663	1.600	1.529	1.597 ± 0.067	1W/2nd	2.672	2.913	2.984	2.856 ± 0.164
7	3W/2nd	1.342	1.426	1.588	1.452 ± 0.125	2W/2nd	2.715	2.785	2.854	2.785 ± 0.070
8	4W/2nd	1.060	1.264	1.326	1.217 ± 0.139	3W/2nd	2.588	2.476	2.387	2.484 ± 0.101
9	5W/2nd	0.923	1.028	1.122	1.024 ± 0.100	4W/2nd	2.275	2.441	2.194	2.303 ± 0.126
10	6W/2nd	0.771	0.878	1.067	0.905 ± 0.150	5W/2nd	1.942	2.359	2.008	2.103 ± 0.224
11	7W/2nd	0.722	0.807	0.867	0.799 ± 0.073	6W/2nd	1.859	1.981	1.879	1.906 ± 0.065
12	8W/2nd	0.609	0.719	0.788	0.705 ± 0.090	7W/2nd	1.785	1.771	1.998	1.851 ± 0.127
13	1W/3rd	1.908	1.516	1.237	1.554 ± 0.337	8W/2nd	1.224	1.396	1.916	1.512 ± 0.360
14	2W/3rd	1.797	1.840	1.504	1.714 ± 0.183	9W/2nd	1.333	1.309	1.807	1.483 ± 0.281
15	3W/3rd	1.814	2.040	1.599	1.818 ± 0.221	10W/2nd	1.343	—	1.714	1.529 ± 0.262
16	4W/3rd	1.658	1.889	1.373	1.640 ± 0.258	11W/2nd	1.201	—	1.494	1.348 ± 0.207
17	5W/3rd	1.327	1.520	1.142	1.330 ± 0.189	1W/3rd	2.821	—	3.439	3.130 ± 0.437
18	6W/3rd	1.068	1.436	1.099	1.201 ± 0.204	2W/3rd	3.029	—	3.377	3.203 ± 0.246
19	7W/3rd	0.950	1.311	0.747	1.003 ± 0.286	3W/3rd	2.907	—	3.129	3.018 ± 0.157
20	8W/3rd	0.806	1.140	0.644	0.863 ± 0.253	4W/3rd	2.447	—	2.952	2.700 ± 0.357
21	1W/4th	1.575	2.243	1.478	1.765 ± 0.417	5W/3rd	2.228	—	2.842	2.535 ± 0.434
22	2W/4th	1.807	2.529	1.945	2.094 ± 0.383	6W/3rd	1.884	—	2.629	2.257 ± 0.527
23	3W/4th	1.482	2.323	1.913	1.906 ± 0.421	7W/3rd	1.600	—	2.453	2.027 ± 0.603
24	4W/4th	1.260	2.118	1.605	1.661 ± 0.432	8W/3rd	1.462	—	2.169	1.816 ± 0.500
25	5W/4th	1.139	1.911	1.568	1.539 ± 0.387	9W/3rd	1.176	—	1.987	1.582 ± 0.573
26	6W/4th	1.018	1.680	1.398	1.365 ± 0.332	10W/3rd	1.123	—	1.867	1.495 ± 0.526
27	7W/4th	0.818	1.618	1.222	1.219 ± 0.400	11W/3rd	0.928	—	1.670	1.299 ± 0.525
28	8W/4th	0.705	1.532	1.077	1.105 ± 0.414	12W/3rd	0.745	—	1.505	1.125 ± 0.537

Anti-HEV IgG levels are expressed as OD450 values. Data are presented as the mean ± SD. Pig No. II-2-21 in the three-dose group died of pneumonia at 14 weeks of age; therefore, data from two pigs were available from 15 weeks of age onward. Pre-imm., before immunization; W, week(s) after the indicated vaccination; SD, standard deviation. —, no sample available.
